# Supplementary material for: Association between Taxonomic Composition of Gut Microbiota and Host Single Nucleotide Polymorphisms in Crohn’s Disease Patients from Russia
Source: Int J Mol Sci. 2023 Apr 28;24(9):7998. doi: 10.3390/ijms24097998 (PMC10178390; doi:10.3390/ijms24097998)
Supplement: Supplementary file 1 [file ijms-24-07998-s001.zip › ijms-2361326-supplementary.pdf]

Supplementary Table S1. Statistically significant differences in the gut microbiota of CD patients with different community types.

	**mean±SD**		**P value (Kruskal-Wallis test)**
	**Community type I**	**Community type II**	
**Diversity**			
N OTUs	1576.34±577.62	679.83±319.72	<0.001
Shannon's index	7.09±0.76	5.33±0.99	<0.001
**Phylum (%)**			
Bacteroidetes	22.60±15.96	14.86±19.21	0.002
Fusobacteria	0.03±0.15	1.42±3.13	0.016
Proteobacteria	3.43±4.13	13.96±15.23	<0.001
Tenericutes	0.22±0.66	0.01±0.06	<0.001
Verrucomicrobia	0.99±2.63	2.29±9.04	0.004
**Family (%)**			
Corynebacteriaceae	0.002±0.005	0.02±0.05	0.010
Micrococcaceae	0.01±0.02	0.08±0.14	<0.001
Nocardiaceae	0.00±0.00	0.002±0.01	0.021
Coriobacteriaceae	2.12±1.87	1.68±3.00	<0.001
o__Bacteroidales;f__	0.34±1.67	0.14±0.79	<0.001
Bacteroidaceae	13.86±12.46	10.42±15.41	0.002
Porphyromonadaceae	1.34±1.63	1.46±3.05	<0.001
Prevotellaceae	3.80±7.64	1.05±2.37	0.001
Rikenellaceae	1.49±1.82	0.54±1.05	<0.001
[Barnesiellaceae]	0.61±1.44	0.26±0.88	<0.001
[Odoribacteraceae]	0.33±0.46	0.26±0.53	0.001
[Paraprevotellaceae]	0.45±0.68	0.58±2.10	<0.001
Bacillaceae	0.01±0.01	0.04±0.10	0.001
Staphylococcaceae	0.01±0.03	0.01±0.03	0.006
Gemellaceae	0.01±0.03	0.06±0.16	0.016
Aerococcaceae	0.0002±0.001	0.003±0.01	0.017

Carnobacteriaceae	0.01±0.02	0.11±0.32	<0.001
Enterococcaceae	0.30±0.95	4.89±8.80	0.012
Streptococcaceae	2.86±4.15	8.10±13.92	0.024
o__Clostridiales;Other	1.06±1.16	0.30±0.46	<0.001
o__Clostridiales;f__	3.36±2.61	0.80±1.20	<0.001
Christensenellaceae	0.47±1.18	0.06±0.33	<0.001
Dehalobacteriaceae	0.01±0.01	0.002±0.01	<0.001
Lachnospiraceae	24.33±10.84	20.82±21.49	0.015
Peptococcaceae	0.05±0.14	0.001±0.002	<0.001
Ruminococcaceae	23.58±9.56	6.46±8.27	<0.001
[Mogibacteriaceae]	0.18±0.23	0.05±0.09	<0.001
Erysipelotrichaceae	2.85±2.82	1.90±3.08	0.001
Leptotrichiaceae	0.0001±0.001	0.07±0.41	0.013
Victivallaceae	0.01±0.03	0.002±0.01	0.021
c__Alphaproteobacteria;o__RF32;f__	0.12±0.34	0.03±0.11	<0.001
Alcaligenaceae	0.49±0.68	1.10±2.14	0.034
Desulfovibrionaceae	0.18±0.22	0.26±0.78	<0.001
Enterobacteriaceae	2.23±4.00	10.29±14.62	<0.001
Legionellaceae	0.00±0.00	0.002±0.01	0.021
Halomonadaceae	0.0002±0.001	0.001±0.004	0.049
Pseudomonadaceae	0.001±0.002	0.003±0.01	0.047
Anaeroplasmataceae	0.14±0.64	0.01±0.06	0.001
[Cerasicoccaceae]	0.02±0.09	0.0001±0.001	0.027
Verrucomicrobiaceae	0.97±2.63	2.29±9.04	0.010

Supplementary Table S2. Inclusion/exclusion criteria for CD patients and healthy subjects.

Inclusion criteria for CD patients:

- m/f, 18-60 years old
- CD diagnosis established by clinical and endoscopic evidence, corroborated by histopathological findings

Exclusion criteria for CD patients:

- Presence of concomitant diseases/conditions which can lead to sufficient changes of the gut microbiota (e.g., oncological pathology, and prior GI surgery)
- The subject has identified congenital or acquired immunodeficiency
- Extensive colonic resection, or ≥3 small bowel resections, or short bowel syndrome. Ileostomy or colostomy
- The subject has a history or evidence of colonic mucosal dysplasia
- *C. difficile* infection or clinically significant infections, including viral hepatitis, tuberculosis, pneumonia
- Intake of prebiotics or/and probiotics within 3 months prior to the visit for biosample collection; antibiotics within 6 months prior to the visit for biosample collection
- Pregnancy or breastfeeding

Inclusion criteria for healthy volunteers:

- m/f, 18-60 years old

Exclusion criteria for healthy volunteers:

- Presence of concomitant diseases/conditions which can lead to sufficient changes of the gut microbiota (e.g., oncological pathology, and prior GI surgery)
- Presence of chronic decompensated cardiovascular, respiratory, liver, kidney, or endocrine diseases
- The subject has identified congenital or acquired immunodeficiency
- *C. difficile* infection or clinically significant infections, including viral hepatitis, tuberculosis, pneumonia
- Presence of diarrhea (stool frequency more than 3 times a day) within at least 3 consecutive days during the last month
- Intake of prebiotics or/and probiotics within 3 months prior to the visit for biosample collection; antibiotics within 6 months prior to the visit for biosample collection
- Pregnancy or breastfeeding

Supplementary Table S3. Studied SNPs

#	SNP	Gene	PCR primers
1	rs2241880 [110–112]	*ATG16L1*	5′-AGGAGACGCTCTGTCTCTTCC-3′
			5′-CACTTCTTTACCAGAACCAGGATG-3′
2	Rs9858542 [27–29]	*BSN*	5′-GGAGAGCGTGTTCTCCAAGAT-3′
			5′-CCAGTTGCAGGCAGTGCATA-3′
3	rs6908425 [113,114]	*CDKAL1*	5′-GGTGACACCCACAAAAGAAAAAGA-3′
			5′-TGCCCTAAGCTAGGGGTTAC-3′
4	rs6596075 [115,116]	*IBD5*	5′-GACCTGAAGAGCTCTGCCTTCAAA-3′
			5′-TCACTGAGGTTCAGTCACCAGC-3′
5	rs11805303 [117,118]	*IL23R*	5′-TGTATGCTTGCAAACAGAGAACT-3′
			5′-AGATTCCTCTTAGTCGGAGCTTTG-3′
6	rs1004819 [119–121]	*IL23R*	5′-CTGGATTGCACTGACCTGCT-3′
			5′-GCACAAGCATTCTAGGACCC-3′
7	rs10489629 [122,123]	*IL23R*	5′-AAGCTTGTTTGATTATGATGTCAGC-3′
			5′-GTCATGCCCAGTTTCCGCTT-3′
8	rs11209026 [111,124]	*IL23R*	5′-TGTCAATTCTTTCTTTGATTGGGAT-3′
			5′-AGAAATTCTGCAAAAACCTACCC-3′
9	rs2522057 [125]	*IRF1-AS1*	5′-TTCATTCATGTCCCTGACACA-3′
			5′-TGCTCACATCTGCCATCTCA-3′
10	rs13361189 [126,127]	*IRGM*	5′-TCTCTCCTCCAAGGCTCCTC-3′
			5′-CCCTTTCTAAACTGTACCCGC-3′
11	rs4958847 [128,129]	*IRGM*	5′-CCTCTCACTGGGAGAAGCTTTA-3′
			5′-TGCATCACAATTTTCTCCTGTTAGT-3′
12	rs2274910 [130]	*ITLN1*	5′-GGAGCCTTTAGGCCATGTCTC-3′
			5′-CAGTGCGGAGAAAATACAGGC-3′
13	Rs1793004 [33,97]	*NELL1*	5′-TGGATCAGGGCCACAATCTT-3′
			5′-TTATGAAGAAACTGGGTTTCCAAGC-3′
14	rs2836878 [131–134]	*PSMG1*	5′-CCAGGCACAAGGACTCTTGA-3′
			5′-AGGCTGGGAGAGGAGATGAG-3′
15	rs1992662 [97]	*PTGER4*	5′-TGCACATTGTGAGCACACATAAA-3′

			5′-TGGTTTCCTTCTATACATATCCGT-3′
16	rs8111071 [135]	*RSPH6A*	5′-CACGCATGACCCTGCAATAC-3′
			5′-TCTGTTGAACCAATGAATGGATGG-3′
17	Rs2631367 [136–138]	*SLC22A5*	5′-CGCTCTGCCTGCCAGC-3′
			5′-GCGGCTGGCCTTACATAGG-3′
18	Rs3816769 [30,31]	*STAT3*	5′-GTCCACTGATAGGAAGATGACTCT-3′
			5′-CTGGTCTTGCAGGGAAGCTA-3′
19	rs7753394 [139,140]	*TNFAIP3*	5′-ATCTCTCACCCTTCTCCTGAAAGA-3′
			5′-GCCCCACATGGTGTAATTTATGC-3′
20	rs1456893 [141]	intergen	5′-ATGTGCCTCTGACCATGACA-3′
			5′-TGCCATGACTAGCTTCTAGGT-3′
21	rs224136 [142,143]	intergen	5′-TAATAGGGCTGTTCACAACTGGA-3′
			5′-ATTCATCCAAGTGCCCAAGTGA-3′
22	rs6601764 [144]	intergen	5′-GGGGGAAGTAGAGAGTTGGAC-3′
			5′-GGAGCATTCAAGTGTGCTTACA-3′
23	rs7807268 [145,146]	intergen	5′-GGAAGGGTGATTTCCTCCTGTT-3′
			5′-CCCTTCGCTACAGACATTGG-3′
24	rs12037606 [147,148]	intergen	5′-TGACAAGCTTTATCACAGCTGATT-3′
			5′-TGCCCTAAGCTAGGGGTTAC-3′
